# Are prenatal anxiety or depression symptoms associated with asthma or atopic diseases throughout the offspring’s childhood? An updated systematic review and meta-analysis

**DOI:** 10.1186/s12884-021-03909-z

**Published:** 2021-06-22

**Authors:** Shuguang Chen, Sheng Chen

**Affiliations:** 1 Department of Dermatology, Southwest Hospital, Third Military M, edical University, Chongqing, China; 2 Department of Pediatrics, Southwest Hospital, Third Military Medical University, Chongqing, 400030 China

**Keywords:** Children, Asthma, Atopic dermatitis, Depression, Anxiety, Pregnancy

## Abstract

**Background:**

Asthma is the most common respiratory disease among children, while atopic diseases such as atopic dermatitis affect about 20% of infants under 2 years of age. Studies suggested that these conditions might be related to prenatal depression or anxiety. This study aimed to explore the association between prenatal mental disorders and childhood asthma or atopic disease in a systematic review and meta-analysis.

**Methods:**

PubMed, Embase, and the Cochrane Library were searched up to May 2020. The primary outcome was childhood asthma and childhood atopic dermatitis. Random-effects models were used because of high heterogeneity indicated by *I*^2^ > 50% and Q-test *P* < 0.10.

**Results:**

A total of 598 studies were initially identified, but nine studies met the inclusion criteria. Prenatal mental disorder was associated with childhood asthma (*n* = 6 studies; ES = 1.146, 95%CI: 1.054–1.245, *P* = 0.001; *I*^2^ = 93.5%, P_heterogeneity_ < 0.001) whereas no significant association was found for childhood atopic dermatitis (*n* = 4 studies; ES = 1.211, 95%CI: 0.982–1.494, *P* = 0.073; *I*^2^ = 78.5%, P_heterogeneity_ < 0.001). Childhood asthma seems to be related more to depression (*n* = 1 study; ES = 1.170, 95%CI: 1.061–1.291, *P* = 0.002) and anxiety/depression (*n* = 4 studies; ES = 1.157, 95%CI: 1.050–1.275, *P* = 0.073; *I*^2^ = 95.3%, P_heterogeneity_ < 0.001).

**Conclusion:**

This meta-analysis demonstrated that prenatal mental disorders increase the risk of childhood asthma. We limited the included samples to pregnant women to investigate the association between prenatal psychological factors and offspring’s physical health. Future studies should include large high-quality cohort studies to investigate the behavioral, environmental, and genetic causes for this association.

**Supplementary Information:**

The online version contains supplementary material available at 10.1186/s12884-021-03909-z.

## Background

In terms of the developmental origins of health and disease, there is evidence that adverse early-life exposure associated with maternal psychiatric diseases can alter the immune system and exacerbate the risk of asthma and atopic diseases such as atopic dermatitis (AD) [1–4]. Asthma is one of the most common respiratory diseases that seriously affects children’s physical and mental health, as recognized by the World Health Organization [5, 6]. Recent studies have investigated the nature and mechanism of disease at the earliest stage in the first few years of life [7–9]. Psychological and emotional factors are considered to trigger the exacerbation of asthma, and emotional stimulation has been proved to lead to the increase of respiratory resistance in asthma [10]. AD, also called atopic eczema, affects many children, especially infants under 2 years old. Some studies showed that the prevalence of AD in children under 2 years old is as high as 20% and reported a two- to three-fold increase in prevalence in the last 30 years [11]. There is evidence for a causal link between maternal mental disorder and AD, and a shared genetic pathway contributes to this familial liability [12].

A meta-analysis of 41 studies carried out in 2019 revealed a significant influence of parental (irrespective of maternal/paternal) mental diseases and childhood asthma but no association with AD [13]. In the previous analysis, multiple childhood physical disorders were investigated, and asthma and AD were only two of them, and not all included studies examined these two diseases together. Besides, the meta-analysis examined parental mental disorders, not exclusively the mothers’, which could make more sense biologically because of possible transplacental influence from the mother’s hormones [14, 15]. A new study was published in 2019 and reported that maternal mental disorders are not associated with offspring asthma, whereas low job control might be a more relevant risk factor [16]. Although the association was significant, the odds of environmental effects on the periods between delivery and diagnosis of asthma or AD still cannot be ignored.

Therefore, it is still unclear whether childhood asthma or AD is related to maternal mental diseases. We sought to undertake an updated meta-analysis and systematic review of all studies investigating the prenatal impact of anxiety and depression on childhood asthma and AD.

## Methods

The Preferred Reporting Items for Systematic Reviews and Meta-Analyses (PRISMA) guidelines were implemented for this systematic review and meta-analysis [17]. We searched for relevant articles using the PICO principle, followed by screening based on the inclusion and exclusion criteria.

### Eligibility criteria

We included observational studies that examined the associations between prenatal mental diseases (depression and anxiety) and asthma and AD, with language limited to English. The participants were children and their biological mothers who undertook an intentional study with medical records access. The analysis was based on mothers with prenatal anxiety or depression (exposure) vs. mothers without prenatal anxiety or depression (non-exposure).

### Search strategy

For this systematic review and meta-analysis, we searched PubMed, Embase, and the Cochrane Library for studies published up to May 2020 with language and article type restriction. Supplementary Table 2 presents the search strategies.

### Data extraction

The data were extracted independently by two investigators. The study characteristics (authors, year of publication, country, study design, and sample size), treatment parameters (exposure of the mother during pregnancy, questionnaire for the diagnosis of exposure, age of children when the study was taken, the outcome measurement, the covariates, and the effect size were extracted from the included studies. Disagreements were resolved by discussion.

### Data synthesis

In this meta-analysis, we extracted the adjusted odds ratios (aOR) from each included study unless we determined any additional variables in the causal relationship between exposure and outcome. If aOR were not available, the crude ORs were extracted. When studies showed multiple exposures with multiple impact sizes, we reported only those exposures that the investigator considered the most severe and chronic. When a study reported multiple results separately, we extracted two results for different analyses. When a study reported only analyses of different asthma types, such as early or late-onset transient asthma, we treated the effect size as two independent outcomes. When a single study was available, the effect sizes of that study were presented.

### Quality of the evidence

We assessed the cohort or case–control studies using the Newcastle–Ottawa Scale (NOS) [18]. Quality assessment was evaluated independently by two reviewers. The discrepancies in the assessment were resolved through discussion until a consensus was reached.

### Statistical analysis

STATA SE 14.0 (Stata Corp., College Station, Texas, USA) was used for all analyses. The studies were grouped by types of outcomes. The comparison between results was employed effect and corresponding 95% confidence interval (CI) for each group. We used Cochran’s Q test and I^2^ index to calculate statistical heterogeneity, for which high heterogeneity was defined as I^2^ > 50% and P < 0.10 in the Q-test [19]. A random-effect model was chosen for high heterogeneity. P-values < 0.05 were considered statistically significant. We planned to conduct two sensitivity analyses, including removing the poor-quality studies ranked by determining the meta-analysis’s robustness, including the estimated comparison between the type of exposure, study design, and sample collection continent. We did not estimate the potential publication bias with funnel plots for sensitivity analysis because the number of studies included in the meta-analysis was less than 10. For outcomes with less than 10 studies, the funnel plots and Egger’s test could yield misleading results and were not recommended [20].

## Results

### Study Selection

**Fig. 1:**
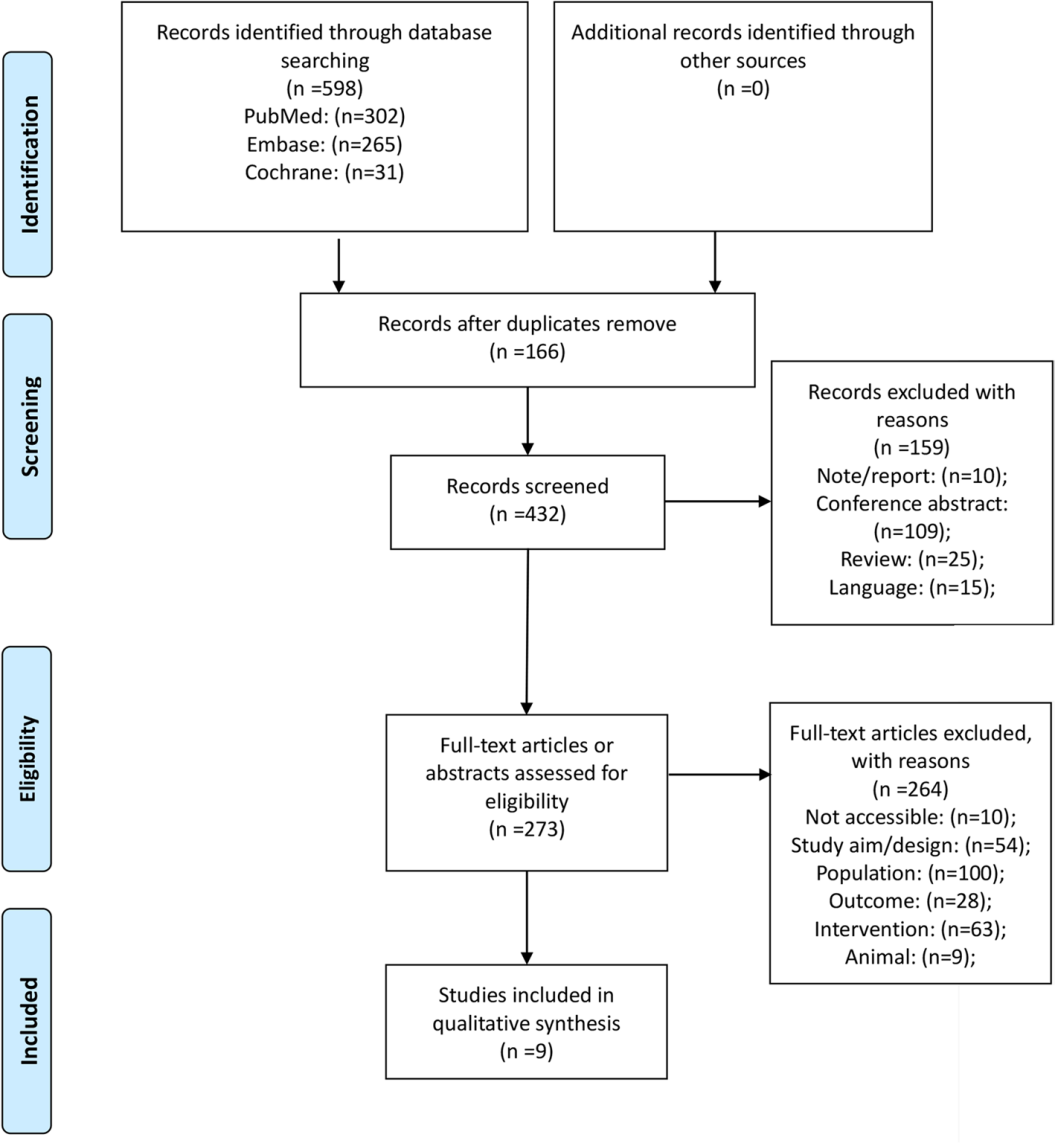
Flow chart of the study selection

Figure 1 shows the study selection procedure and the reasons for exclusion. A total of 598 studies were retrieved from PubMed (*n* = 302), Embase (*n* = 265), and the Cochrane Library (*n* = 31). After removing the duplicates, 432 articles were examined. Ten articles were excluded because of being notes/reports, 109 articles were excluded because they were conference abstracts, 25 articles were excluded because of being reviews, and 15 articles were excluded because of the language. Then, 273 articles were left for full-text screening and 264 were excluded because of being not accessible (*n* = 10), study aim/design (*n* = 54), population (*n* = 100), outcome (*n* = 28), intervention (*n* = 63), and animal (*n* = 9). No additional records were identified through other sources. Hence, nine observational studies entered our final model [10, 12, 16, 21–26].

### Characteristics of the included studies

Table 1 presents the included studies [10, 12, 16, 21–26]. There were four prospective cohort studies [7, 18–20], four retrospective cohort studies [12, 16, 24, 25], and one case–control study [26]. Most studies were from Europe [10, 12, 16, 21, 23, 26], and the others were from America [22, 24, 25]. The studies included 982,942 patients. The studies investigated different exposures, including depression, anxiety, depression or anxiety, mental health service use, prenatal distress, and negative life events. Studies were grouped into the categories according to their outcomes: five for asthma [10, 12, 16, 21, 24], three for AD [22, 23, 26], and one for asthma and AD [25]. To ensure the reliability and quality control of this meta-analysis, we scored each study using the NOS criteria, and studies with more than 5 stars were included in the meta-analysis. After evaluation, six studies scored 9 stars [10, 12, 21, 23, 25, 26], and three studies scored 8 stars [16, 22, 24] (Supplementary Table 1a). Letourneau et al. [22] examined infants of only 18 months of age, and Liu et al. [16] examined children when they were 0–6 years of age; both studies examined their population before the usual onset age for asthma and AD, possibly explaining their lower quality, at least in part. Radhakrishnan et al. defined exposure as any use of mental health service, including a wide variety of mental illnesses besides anxiety and depression [24].

**Table 1:** Literature search and study characteristic

Author, Year	Country	Study Design	N	Exposure (age measured)	Diagnosis of exposure	Outcome and age measured of the outcome	Effect measure	Covariates
Cookson, 2009 [10]	UK, Europe	Prospective cohort study	5810	Anxiety symptom (32 weeks of gestation)	Crown-Crisp index	Asthma (7.5-y)	aOR = 1.03 (0.86,1.23)	Partner's self-reported anxiety symptom scores during pregnancy
Magnus, 2017 [21]	Norway, Europe	Prospective cohort study	63,626	Major depression (30 weeks pregnancy)	SCL-5	Asthma (7-y)	aOR = 1.17 (1.06,1.29)	Maternal age, parity, education, pregnancy body mass index, smoking during pregnancy, and history of asthma
Letourneau, 2017 [22]	Canada, North America	Prospective cohort study	242	Anxiety (32–40 weeks of gestation)	EDS, SCL-90-R	AD (18-month)	aOR = 2.78 (1.04,7.39)	Maternal unresponsiveness and controlling, postnatal depression, social support and anxiety, pregnancy specific anxiety, maternal asthma
Elbert, 2017 [23]	Netherlands, Europe	Prospective cohort study	5205	Depression (2nd trimester of pregnancy)	Brief Symptom Inventory	AD (9-10y)	1. inhalant aOR = 2.07 (1.43,2.97) 2. food aOR = 0.75 (0.29,0.97)	Maternal age at enrollment, education, ethnic origin, parity, pet keeping, BMI at enrollment, smoking and history of allergy, eczema or asthma, and child's sex, gestational age, birth weight, child's ever breastfeeding and day care attendance
Brew, 2018 [12]	Sweden, Europe	Retrospective cohort study	360,526	Depression or anxiety (continuously through preconception, pregnancy)	SCARED, SMFQ	Asthma (5-y)	aOR = 1.44 (1.34,1.56)	Sex, gestational age, birthweight, maternal age, parental country of birth, atopic status of twin 2
Liu, 2019 [16]	Denmar, Europe	Retrospective cohort study	547,533	Negative life events (1 year before conception until delivery)	ICD (10th revision)	Asthma (0 to 6-year)	1. Early-onset transient asthma aPR = 1.02 (0.99,1.06) 2. Early-onset persistent asthma aPR = 1.04 (0.99,1.08) 3. Late-onset asthma aPR = 0.99 (0.93–1.05)	Maternal age at delivery, education at conception, smoking during pregnancy, parity, comorbidity before delivery, parental atopic status, calendar year of birth, negative life events, job demands, and job control
Radhakrishnan, 2018 [24]	Canada, North America	Retrospective cohort study	122,333	Mental health service use (during pregnancy)	NA	Asthma (12-y)	aOR = 1.16 (1.12,1.20)	Maternal history of asthma, the child’s socioeconomic status using neighborhood income quintile as a proxy, urban versus rural residence at birth, sex, low birthweight, and the presence of childhood comorbid illnesses
van der leek, 2020 [25]	Canada, North America	Retrospective cohort study	9995	Maternal distress (both pre and postnatal)	ICD (9th revision)	AD, Asthma (5-, 7-year)	1. AD: aOR = 1.27 (1.11, 1.46) 2.Asthma: aOR = 1.57 (1.29,1.91)	Preterm birth, maternal age, atopy status, urban residence, infant sex and antibiotic treatment
Hamann, 2018 [26]	Denmark, Europe	Case–control	94,622	Depression (during pregnancy)	HAMD	AD (before 5-y)	1. Compared to general population: aOR = 1.12 (0.97, 1.29) 2. Compared to pediatric hospital/clinic population: aOR = 0.91 (0.79, 1.05)	Age, sex, parental AD, and socioeconomic position

*ICD* International Classification of Disease, *SCL-5* 5-item symptom checklist, *SCARED* Screen for Child Anxiety Related Emotional Disorders, *SMFQ* Shortened Mood and Feelings, *EDS* Edinburgh depression scale, *aOR* adjusted odds ratio, *AD* Atopic dermatitis

### Effect of prenatal depression on childhood asthma

**Fig. 2:**
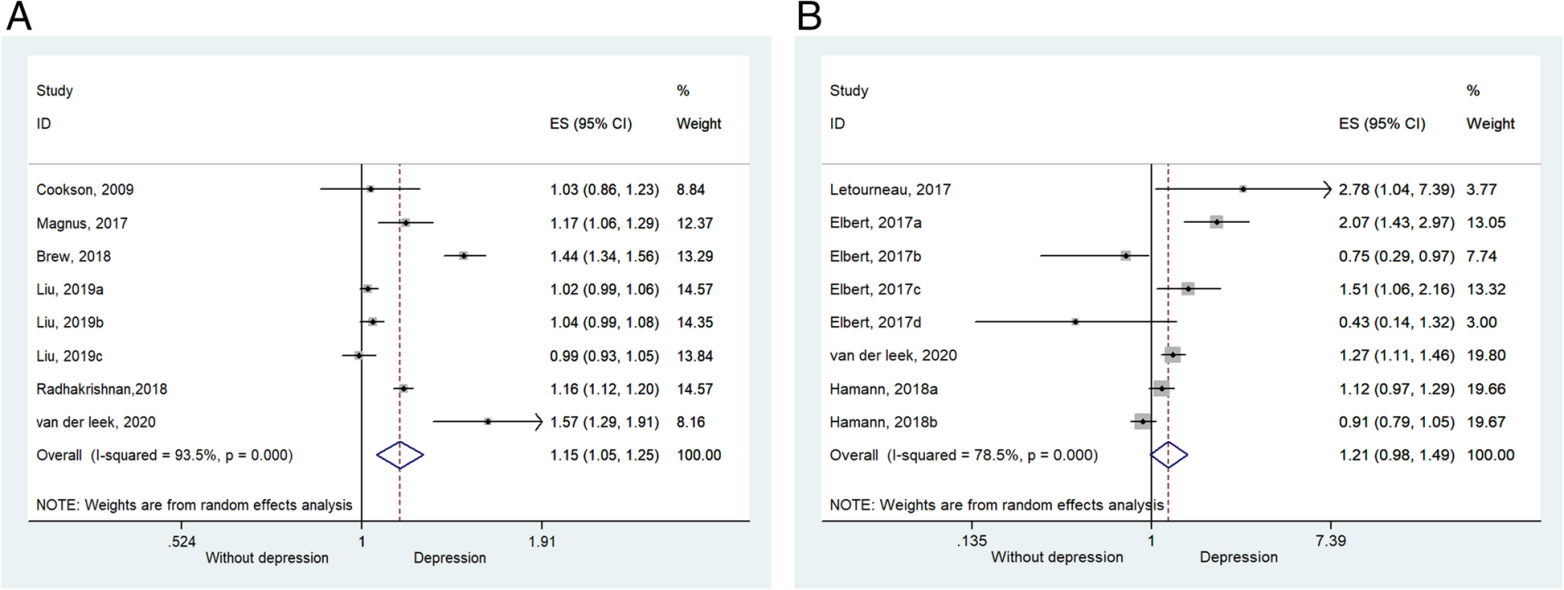
**A** Forest plot of asthma. Children who were born to mothers with prenatal mental illness had increased odds of developing asthma. **B**. Forest plot of AD. The impact of prenatal mental illness was not significant for childhood AD

Six studies (eight datasets) [10, 12, 16, 21, 24, 25] could be included for the meta-analysis of prenatal depression on childhood asthma. Compared with the non-exposure group (maternal without depression), the results showed that prenatal depression influenced childhood asthma (ES = 1.146, 95%CI: 1.054–1.245, *P* = 0.001; *I*^2^ = 93.5%, P_heterogeneity_ < 0.001) (Fig. 2A and Table 2).

**Table 2:** Treatment vs. Control for Asthma

	N	ES (95%CI)	P	I-square, %	P (Heterogeneity)
Asthma	8	1.146(1.054,1.245)	0.001	93.5	< 0.001
Anxiety	1	1.030(0.861,1.232)	0.746		
Depression	1	1.170(1.061,1.291)	0.002		
Anxiety/depression	6	1.157(1.050,1.275)	0.003	95.3	< 0.001
Prospective cohort	2	1.123(1.000,1.262)	0.051	33.2	0.221
Retrospective cohort	6	1.157(1.050,1.275)	0.003	95.3	< 0.001
Europe	6	1.106(1.001,1.221)	0.047	93.5	< 0.001
North America	2	1.328(0.989,1.784)	0.059	88.7	0.003

### Effect of prenatal depression on childhood AD

Four studies (eight datasets) [22, 23, 25, 26] could be included for the meta-analysis of prenatal depression on childhood AD. The results indicated that there was no statistically significant difference between the non-exposure and exposure groups, which means that prenatal depression may not influence childhood AD (ES = 1.211, 95%CI: 0.982–1.494, P = 0.073; I^2^ = 78.5%, P_heterogeneity_ < 0.001) (Fig. 2B and Table 3).

**Table 3:** Treatment vs. Control for AD

	N	ES (95%CI)	P	I-square, %	P (Heterogeneity)
AD	8	1.211(0.982,1.494)	0.073	78.5	< 0.001
Anxiety	3	1.305(0.576,2.959)	0.523	68	0.044
Depression	4	1.138(0.847,1.528)	0.391	84.3	< 0.001
Anxiety/depression	1	1.270(1.107,1.457)	0.001		
Prospective cohort	5	1.329(0.816,2.164)	0.253	72.1	0.006
Retrospective cohort	1	1.270(1.107,1.457)	0.001		
Case–control	2	1.010(0.824,1.237)	0.927	75.5	0.043
Europe	2	1.144(0.876,1.494)	0.322	80.3	< 0.001
North America	6	1.607(0.795,3.248)	0.187	58.4	0.121

### Subgroup analyses of childhood asthma

**Fig. 3:**
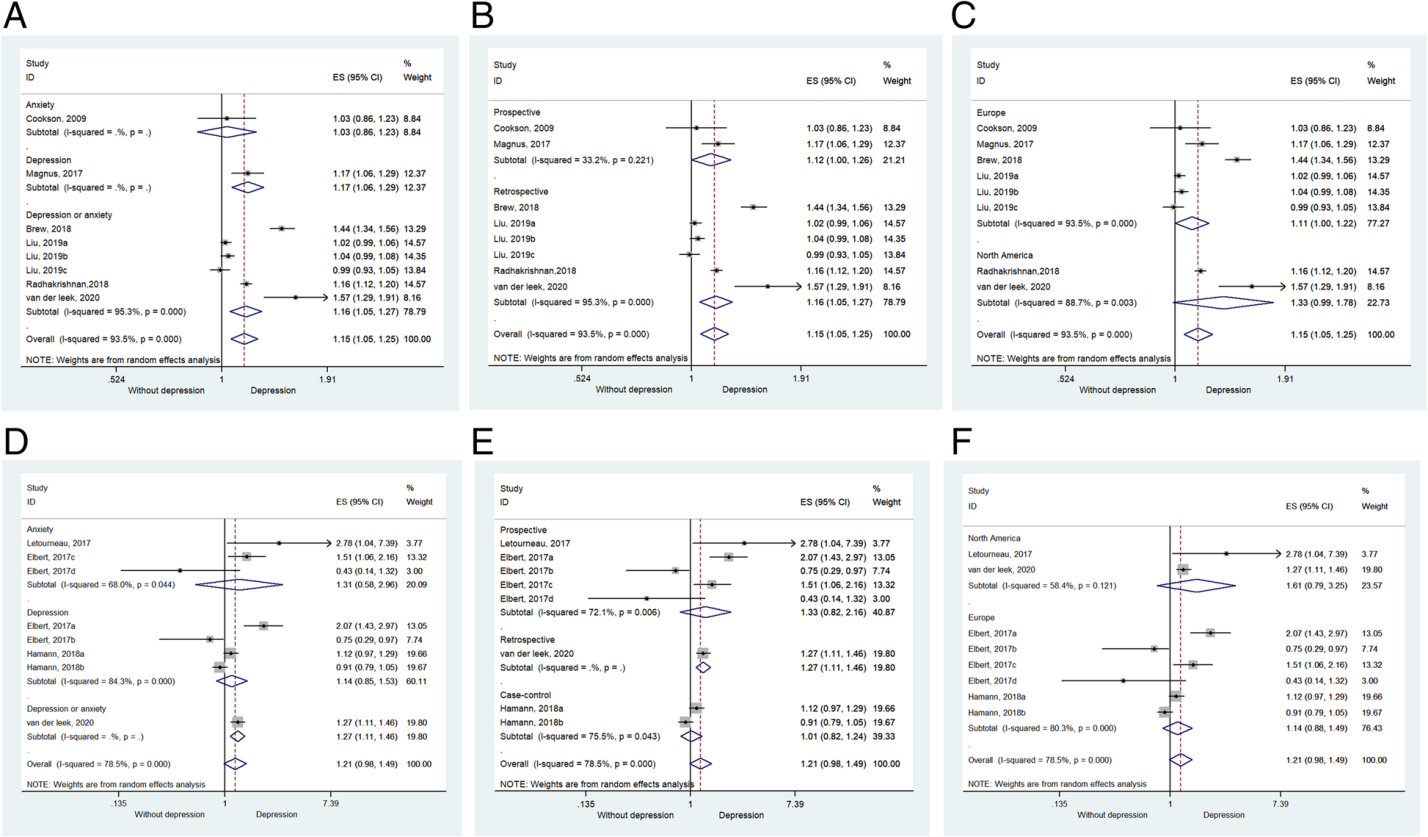
**A**. Forest plot of asthma by type of exposure. The association between prenatal anxiety and childhood asthma was ambiguous. But clear evidence for the link between prenatal depression, as well as anxiety/depression, with asthma. **B**. Forest plot of AD by type of exposure. **C**. Forest plot of asthma by type of study design. A retrospective study concluded the significant association between prenatal mental illness and childhood asthma, whereas a prospective cohort study stands reversely (*P* = 0.051). **D** Forest plot of AD by type of study design. Only a retrospective cohort study stands for a positive association between prenatal mental illness and childhood AD, whereas others not. **E** Forest plot of asthma by where the sample was collected. F). Forest plot of AD by where the sample was collected

Only one study [10] examined the association between prenatal anxiety and childhood asthma (ES = 1.03, 95%CI: 0.86–1.23, *P* = 0.746). Another study examined prenatal depression and childhood asthma [21] and reported a significant association (ES = 1.17, 95%CI: 1.06–1.29, *P* = 0.002). Four studies considered prenatal depression or anxiety [12, 16, 24, 25] and a significant association was observed (ES = 1.16, 95%CI: 1.05–1.27, *P* = 0.003; *I*^2^ = 95.3%, P_heterogeneity_ < 0.001) (Fig. 3A and Table 2).

There were two prospective studies [10, 21] (ES = 1.123, 95%CI: 1.000–1.262, *P* = 0.051; *I*^2^ = 33.2, P_heterogeneity_ = 0.221) that showed a borderline possible association between prenatal mental disorder and childhood asthma, whereas four retrospective studies [12, 16, 24, 25] indicated that childhood asthma was associated with prenatal mental disorder (ES = 1.157, 95%CI: 1.050–1.275, *P* = 0.003; *I*^2^ = 95.3, P_heterogeneity_ < 0.001) (Fig. 3B and Table 2). Four studies from Europe [10, 12, 16, 21] showed that childhood asthma was associated with prenatal mental disorder (ES = 1.106, 95%CI: 1.001–1.221, *P* = 0.047; *I*^2^ = 93.5, P_heterogeneity_ < 0.001), but two studies from North America [24, 25] suggested the opposite conclusion (ES = 1.328, 95%CI: 0.989–1.784, *P* = 0.059; *I*^2^ = 88.7, P_heterogeneity_ = 0.003) (Fig. 3C and Table 2).

### Subgroup analyses of childhood AD

Two studies [22, 23] indicated no association between prenatal anxiety and childhood AD (ES = 1.31, 95%CI: 0.58–2.96, *P* = 0.523; *I*^2^ = 68, P_heterogeneity_ = 0.044). Two other studies [23, 26] demonstrated that there was no significant association between prenatal depression and childhood AD (ES = 1.14, 95%CI: 0.85–1.53, *P* = 0.391; *I*^2^ = 84.3, P_heterogeneity_ < 0.001). One study [25] showed that childhood AD was associated with prenatal depression or anxiety (ES = 1.27, 95%CI: 1.11–1.46, *P* = 0.001) (Fig. 3D and Table 3).

Two prospective studies [22, 23] (ES = 1.329, 95%CI: 0.816–2.164, *P* = 0.253; I^2^ = 72.1, P_heterogeneity_ = 0.006) and one case–control study [26] (ES = 1.010, 95%CI: 0.824–1.237, *P* = 0.927; *I*^2^ = 75.5, P_heterogeneity_ = 0.043) showed no association between prenatal mental disorder and childhood AD, whereas only one retrospective cohort study [25] showed the opposite (ES = 1.27, 95%CI: 1.11–1.46, *P* = 0.391; *I*^2^ = 84.3, P_heterogeneity_ < 0.001) (Fig. 3E and Table 3).

Two studies from Europe [23, 26] (ES = 1.144, 95%CI: 0.876–1.494, *P* = 0.322; *I*^2^ = 80.3, P_heterogeneity_ < 0.001) and two studies from North America [22, 25] (ES = 1.607, 95%CI: 0.795–3.248, *P* = 0.187; *I*^2^ = 58.4, P_heterogeneity_ = 0.121) suggested that there was no correlation between childhood AD and prenatal mental disorder (Fig. 3F and Table 2).

### Sensitivity analyses

The sensitivity analyses indicated that publication bias was not significant since no individual study affected the observed result for childhood asthma (Supplementary Fig. 1A) and childhood AD (Supplementary Fig. 1B).

## Discussion

This study aimed to explore the association between prenatal psychiatric disorders and childhood asthma or atopic disease in a systematic review and meta-analysis of nine studies. The results indicate that prenatal mental disorders increase the risk of childhood asthma.

The strengths of this meta-analysis include the large number of patients included. Besides, only maternal exposure was considered. Indeed, even if a child shares the genes from both parents, only the maternal intrauterine environment influences the child. Finally, only studies on anxiety or depression and asthma or AD were included, reducing heterogeneity. The focus of the previous meta-analysis was to explore the effects of parental (both paternal and maternal) mental illnesses on children’s physical health, revealing that prenatal mental disorder contributes to poor fetal growth and further suggesting the impact of maternal mental diseases during pregnancy on children [13]. In the present meta-analysis, attention was paid to the impact of maternal mental health during pregnancy on offspring’s childhood systemic autoimmune diseases. Even though the number of included studies was different from the previous meta-analysis [13], the included sample size that entered the final analysis was similar and could explain the results’ consistency. Future studies should examine the paternal and maternal mental diseases separately [27].

Nevertheless, this meta-analysis has limitations, and the results must be weighed against these limitations. All identified studies were observational and are therefore subject to confounding bias. Moreover, there were several retrospective studies. Factors such as selection bias, recall bias, and information bias were inevitably inherent in our analysis. Although we extracted the adjusted effect sizes for analysis, the covariates of each model were different. Some studies may be subject to over-adjustment, where analyses adjust for variables on the causal pathway between the exposure and the outcome. Although we actively tried to include unpublished research, all the identified studies were from the published literature. Therefore, it might well be that some positive findings are the result of publication bias. Furthermore, some caveats should be considered when interpreting the findings of subgroup analysis of childhood asthma since only Cookson et al. [10] reported an association between prenatal maternal anxiety and asthma, and another study by Magnus et al. [21] examined an association between prenatal maternal depression and childhood asthma.

Some studies have reported that anxiety/stress during pregnancy can aggravate the activation of the hypothalamic–pituitary–adrenal (HPA) axis, leading to the release of cortisol, which the placenta cannot entirely metabolize, and may even promote the release of placental glucocorticoids and crosses to the fetus, in which they can influence the fetal brain development and may result in airway inflammation and hyperresponsiveness [28, 29]. Maternal stress-induced changes in cortisol levels might affect fetal immune regulation and TH2 lymphocyte dominance by directly affecting cytokine production [14]. In human subjects, prenatal mental diseases were associated with changes in the inherent and adaptive immune responses in infants’ umbilical cord blood at high risk for atopic disease [15]. β_2_-adrenoreceptors [30] expressed throughout the body were stimulated by the stress hormone adrenaline [31, 32]. The investigators identified that maternal mental diseases during pregnancy might affect fetal growth, especially low-birth-weight infants with smaller lungs and airways, leading to a high asthma risk [33–35]. Given this wealth of possible mechanisms, focusing on maternal mental disorder’s association during pregnancy and childhood asthma is meaningful. Of course, various genetic factors might be involved in the fetal response to stress hormones [36] or might predispose the child to asthma and AD [37]. These genetic factors should be explored in future studies.

The present study is a systematic review and meta-analysis (which has not been previously prospectively registered) on prenatal anxiety or depression symptoms and childhood asthma or AD. In the nine studies’ final analysis, the relationship between prenatal mental disorder and childhood asthma was statistically significant compared with childhood AD. The present study results are consistent with the previous meta-analysis [13], further confirming that prenatal mental disorders are associated with childhood asthma.

The association between prenatal maternal depression and childhood asthma seems more significant but only included one study that met the criteria [21]. In theory, prospective studies are more reliable than retrospective ones, but the associations were not significant in the included prospective studies [10, 21]. The results showed that asthma was significantly associated with maternal preconception mental status in Europe [10, 12, 16, 21]. Although the combined overall aOR value was statistically significant (*P* = 0.001), the association was not statistically significant for the North American studies (*P* = 0.059) [24, 25]. More adequately designed large-scale prospective studies are needed to provide a decisive answer about the association between maternal prenatal anxiety/depression and childhood asthma.

Although the results of this updated meta-analysis are consistent with the previous one [13], we limited the included studies to pregnant women to investigate the association between prenatal psychological factors and offspring’s physical health. Future research might deepen our understanding of when and how these vulnerable children are at risk of preventable illnesses. The results also highlight the need for investments in the development of prevention programs during pregnancy, targeting maternal mental health promotion. Future studies should include large high-quality cohort studies to investigate the behavioral, environmental, and genetic causes for this association.

## Conclusion

In conclusion, this meta-analysis supports that prenatal mental disorders increase childhood asthma risk, whereas no significant association was found for childhood AD.

## Supplementary Information

**Additional file 1: Supplementary Figure 1.** A). Sensitivity analysis of asthma. Sensitivity analysis suggested the results are robust. B). Sensitivity analysis of AD.

**Additional file 2: Supplementary Table 1a.** NOS criteria for cohort study. **Supplementary Table 1b.** NOS criteria for quality of case-control study.

**Additional file 3: Supplementary Table 2.** Search terms and strategy.

## Data Availability

The datasets used and/or analyzed during the current study are available from the corresponding author on reasonable request.

## Abbreviations

PRISMA: The Preferred Reporting Items for Systematic Reviews and Meta-Analyses
NOS: The Newcastle–Ottawa Scale
CI: Confidence interval
