# Supplementary figures and images for: Are prenatal anxiety or depression symptoms associated with asthma or atopic diseases throughout the offspring’s childhood? An updated systematic review and meta-analysis
Source: BMC Pregnancy Childbirth. 2021 Jun 22;21:435. doi: 10.1186/s12884-021-03909-z (PMC8218439; doi:10.1186/s12884-021-03909-z)

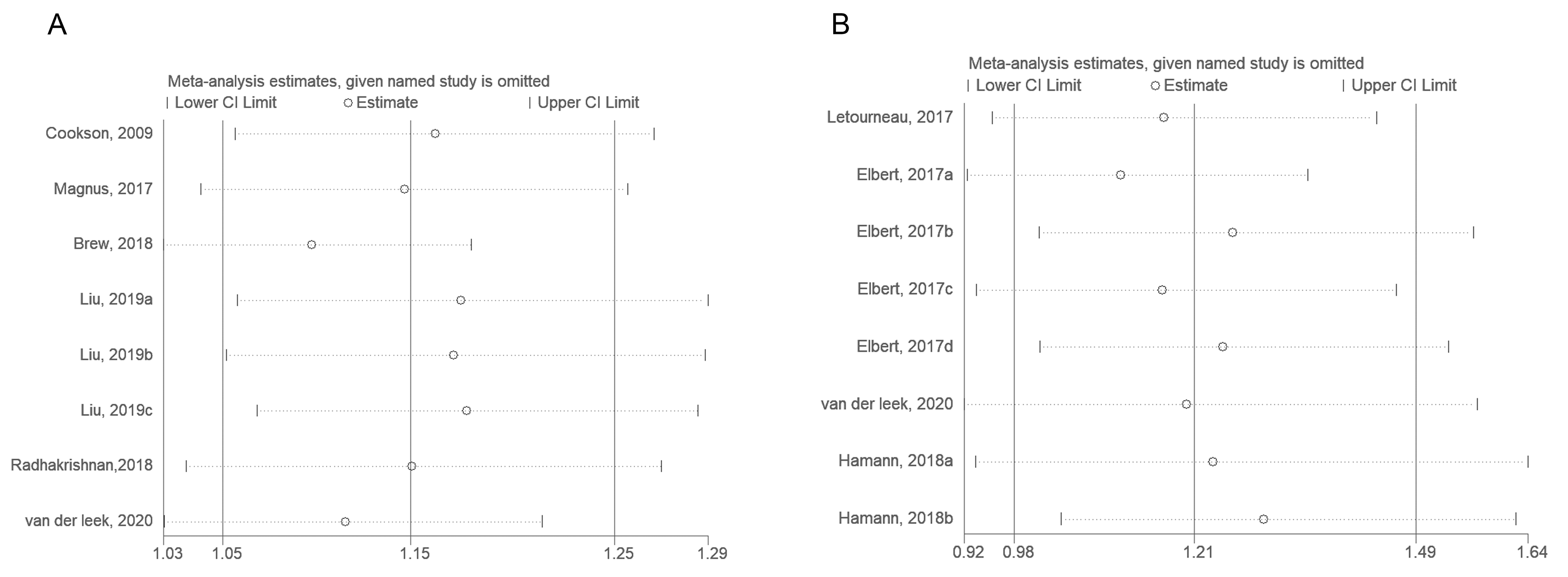

Supplement: Supplementary file 1 — Additional file 1: Supplementary Figure 1. A). Sensitivity analysis of asthma. Sensitivity analysis suggested the results are robust. B). Sensitivity analysis of AD. [file 12884_2021_3909_MOESM1_ESM.tif]
