# Supplementary material for: Are prenatal anxiety or depression symptoms associated with asthma or atopic diseases throughout the offspring’s childhood? An updated systematic review and meta-analysis
Source: BMC Pregnancy Childbirth. 2021 Jun 22;21:435. doi: 10.1186/s12884-021-03909-z (PMC8218439; doi:10.1186/s12884-021-03909-z)
Supplement: Supplementary file 2 — Additional file 2: Supplementary Table 1a. NOS criteria for cohort study. Supplementary Table 1b. NOS criteria for quality of case-control study. [file 12884_2021_3909_MOESM2_ESM.docx]

**Supplementary Table 1a**. NOS criteria for cohort study

| Study | Representativeness of the exposed cohort | Selection of the non-exposed cohort | Ascertainment of exposure | Demonstration that outcome of interest was not present at start of study | Comparability of cohorts on the basis of the design or analysis | Assessment of outcome | Was follow-up long enough for outcomes to occur | Adequacy of follow up of cohorts | Total quality scores |
| --- | --- | --- | --- | --- | --- | --- | --- | --- | --- |
| Cookson, 2009 | ☆ | ☆ | ☆ | ☆ | ☆☆ | ☆ | ☆ | ☆ | 9 |
| Magnus, 2017 | ☆ | ☆ | ☆ | ☆ | ☆☆ | ☆ | ☆ | ☆ | 9 |
| Letourneau, 2017 | ☆ | ☆ | ☆ | ☆ | ☆☆ | ☆ | - | ☆ | 8 |
| Elbert, 2017 | ☆ | ☆ | ☆ | ☆ | ☆☆ | ☆ | ☆ | ☆ | 9 |
| Brew, 2018 | ☆ | ☆ | ☆ | ☆ | ☆☆ | ☆ | ☆ | ☆ | 9 |
| Liu, 2019 | ☆ | ☆ | ☆ | ☆ | ☆☆ | ☆ | - | ☆ | 8 |
| Radhakrishnan,2018 | ☆ | ☆ | - | ☆ | ☆☆ | ☆ | ☆ | ☆ | 8 |
| van der leek, 2020 | ☆ | ☆ | ☆ | ☆ | ☆☆ | ☆ | ☆ | ☆ | 9 |

**Supplementary Table 1b**. NOS criteria for quality of case-control study

| Study | Is the case difinition adequate? | Representativeness of the cases | Selection of controls | Definition of controls | Comparablity of cases and controls on the basis of the design or analysis | Ascertainment of intervention | Same method of acrertainment for cases and controls | Non-response rate | Total quality scores |
| --- | --- | --- | --- | --- | --- | --- | --- | --- | --- |
| Hamann, 2018 | ☆ | ☆ | ☆ | ☆ | ☆☆ | ☆ | ☆ | ☆ | 9 |
