# Supplementary material for: Are prenatal anxiety or depression symptoms associated with asthma or atopic diseases throughout the offspring’s childhood? An updated systematic review and meta-analysis
Source: BMC Pregnancy Childbirth. 2021 Jun 22;21:435. doi: 10.1186/s12884-021-03909-z (PMC8218439; doi:10.1186/s12884-021-03909-z)
Supplement: Supplementary file 3 — Additional file 3: Supplementary Table 2. Search terms and strategy. [file 12884_2021_3909_MOESM3_ESM.docx]

**Supplementary Table 2.** Search terms and strategy.

This appendix provides the questions searched for, an overview of the search strategy, and detailed search terms and logic used in each database (PubMed, Embase and Cochrane).

|  |  | **Search strategy** | **Numbers** |
| --- | --- | --- | --- |
| **PubMed** |  |  |  |
| **Patient** | **#1** | ((((((((child[Title/Abstract]) OR (children[Title/Abstract])) OR (teen*[Title/Abstract])) OR (adolescent[Title/Abstract])) OR (infant[Title/Abstract])) OR (newborn[Title/Abstract])) OR (offspring[Title/Abstract])) OR (childhood[Title/Abstract])) OR (preschool[Title/Abstract]) | 1775670 |
| **Intervention** | **#2** | ((maternal[Title/Abstract]) OR (mother*[Title/Abstract])) AND ((((((distress[Title/Abstract]) OR (Mental Disorder[Title/Abstract])) OR (stress*[Title/Abstract])) OR (Negative Life Events[Title/Abstract])) OR (depression[Title/Abstract])) OR (anxiety[Title/Abstract])) | 42789 |
| **Outcome** | **#3** | (((((((asthma[Title/Abstract]) OR (atopic dermatitis[Title/Abstract])) OR (atopic eczema[Title/Abstract])) OR (atopic sensitization[Title/Abstract])) OR (food allergy[Title/Abstract])) OR (allergic rhinitis[Title/Abstract])) OR (urticaria[Title/Abstract])) OR (anaphylaxis[Title/Abstract]) | 210312 |
| **Study type** | #4 | (((((((((case?control) OR (cohort)) OR (follow?up stud*)) OR (longitudinal stud*)) OR (odds ratio)) OR (relative?risk)) OR (rate?ratio)) OR (hazard ratio)) OR (prospective)) OR (retrospective) | 5185480 |
|  |  | #1 AND #2 AND #3 AND #4 | 302 |
| **Embase** |  |  |  |
| **Patient** | **#1** | child:ti,ab OR children:ti,ab OR teen*:ti,ab OR adolescent:ti,ab OR infant:ti,ab OR newborn:ti,ab OR offspring:ti,ab OR childhood:ti,ab OR preschool:ti,ab | 2214446 |
| **Intervention** | **#2** | maternal:ti,ab OR mother*:ti,ab | 510145 |
|  | **#3** | distress:ti,ab OR 'mental disorder':ti,ab OR stress*:ti,ab OR 'negative life events':ti,ab OR depression:ti,ab OR anxiety:ti,ab | 1671960 |
|  | **#4** | #2 AND #3 | 57261 |
| **Outcome** | **#5** | asthma:ti,ab OR ‘atopic dermatitis’:ti,ab OR ‘atopic eczema’:ti,ab OR ‘atopic sensitization’:ti,ab OR ‘food allergy’:ti,ab OR ‘allergic rhinitis’:ti,ab OR urticaria:ti,ab OR anaphylaxis:ti,ab | 306209 |
| **Study type** | #6 | case?control OR cohort OR ‘follow?up stud*’ OR ‘longitudinal stud*’ OR ‘odds ratio’ OR relative?risk OR rate?ratio OR ‘hazard ratio’ OR prospective OR retrospective | 3096844 |
| **Total** | #7 | #1 AND #4 AND #5 AND #6 | 265 |
| **Cochrane** |  |  |  |
| **Patient** | **#1** | child OR children OR teen* OR adolescent OR infant OR newborn OR offspring OR childhood OR preschool | 259548 |
| **Intervention** | **#2** | (maternal OR mother*) AND (distress OR Mental Disorder OR stress* OR Negative Life Events OR depression OR anxiety) | 6753 |
| **Outcome** | **#3** | asthma OR atopic dermatitis OR atopic eczema OR atopic sensitization OR food allergy OR allergic rhinitis OR urticaria OR anaphylaxis | 46921 |
| **Study type** | #4 | case?control OR cohort OR follow?up stud* OR longitudinal stud* OR odds ratio OR relative?risk OR rate?ratio OR hazard ratio OR prospective OR retrospective | 428045 |
| **Total** | #5 | #1 AND #2 AND #3 AND #4 | 194(31trials) |
